# Role of vaccination in the sustainability of healthcare systems

**DOI:** 10.3402/jmahp.v3.27043

**Published:** 2015-08-12

**Authors:** Nathalie Largeron, Pierre Lévy, Jürgen Wasem, Xavier Bresse

**Affiliations:** 1 Sanofi Pasteur MSD, Lyon, France; 2 LEDa-LEGOS, Université Paris-Dauphine, Paris, France; 3 Institute of Health Care Management and Research, Universität Duisburg, Essen, Germany

**Keywords:** vaccination, healthcare resource use, cancers, costs, nosocomial infections

## Abstract

The use of vaccines to prevent diseases in children, adults, and the elderly results in fewer medical visits, diagnostic tests, treatments, and hospitalisations, which leads to substantial savings in healthcare costs each year in Europe and elsewhere. Vaccines also contribute to reducing resource utilisation by preventing nosocomial infections, such as rotavirus gastroenteritis, which can increase hospital stays by 4–12 days. Vaccination also has an important role in the prevention of cancers with, for example, human papillomavirus or hepatitis B vaccines. Since the financial impact of cancer is high for patients, healthcare systems, and society as a whole, any cases prevented will reduce this impact. Newer vaccines, such as the herpes zoster vaccine, can provide an answer to unmet medical needs by preventing and reducing the severity of shingles and associated post-herpetic neuralgia, which are difficult conditions to treat. Thus, in the context of increasing pressure on healthcare budgets, vaccination can contribute to the sustainability of healthcare systems through reduced and more efficient use of healthcare resources.

In the past decades, vaccination has dramatically reduced the incidence of several infectious diseases that were responsible for much suffering and deaths. The impact of vaccination has been demonstrated by the estimate that during the ‘vaccines for children era’ (1994–2013), the total number prevented by routine childhood vaccinations in the USA was more than 322 million cases of infectious diseases, 21 million hospitalisations, and 731,700 deaths (1). Thus, vaccination has made a substantial contribution to the sustainability of healthcare systems by reducing the burden of frequent infectious diseases and associated resource use. There is an increasing number of vaccines protecting against a range of infections affecting not just children but also adults and the elderly. In this article, we will examine how an effective use of these vaccines can further reduce the burden on healthcare systems by reducing healthcare resource utilisation, preventing severe diseases such as cancers and nosocomial infections, and answering unmet medical needs.

## Reduction of hospitalisations, ambulatory care visits, and medical interventions

### In children

Today, children in Europe routinely receive vaccines that protect them from more than a dozen diseases. As presented in the first article of this special issue, childhood vaccination resulted in a substantial decrease in the incidence of numerous infectious diseases and associated mortality such as diphtheria, tetanus and polio, tuberculosis, pertussis, measles, mumps, and rubella (2). For example, prior to introduction of the conjugate vaccine, Haemophilus influenzae b (Hib) was the leading cause of childhood meningitis, pneumonia, and epiglottitis, causing an estimated 20,000 cases per year in the early 1980s in the United States, mostly in children under 5 years old. Since routine vaccination began between 1980 and 1990, the incidence of Hib diseases has declined by greater than 99%. Similar reductions in disease occurred after introduction of the vaccine in Western Europe and developing countries. These ‘traditional’ vaccines have become the cornerstone of efficient healthcare systems throughout the world and have been shown to be highly cost-saving (2). Since the late 1990s, new vaccines, such as rotavirus (RV), meningococcal, pneumococcal conjugate, or varicella vaccines have become available. Although access to these new vaccines is not homogeneous across Europe, they have been shown to reduce the costs associated with hospitalisations and outpatient visits (Table 1) (3–13).

**Table 1:** Estimated human and economic burden in Europe of some new preventable diseases

	Annual burden before vaccine introduction	Cost per case
Rotavirus gastroenteritis	In Europe in children <5 years (3) ‐ 3.6 million episodes ‐ 87,000 hospital admissions ‐ 700,000 GP consultations ‐ 231 deaths	Societal perspective (including direct medical, direct non-medical, and indirect costs) in 2004–2005 in Belgium, France, Germany, Italy, Spain, Sweden, and UK (5) ‐ From €166 to €473 in the primary care setting ‐ From €334 to €770 in the emergency department setting ‐ From €1,525 to €2,101 in the hospital setting ‐ Mean number of work days lost by parents: 2.3–7.5 days
Meningitis C	In the UK: 955 cases in 1998 (7)	In the UK: £8,413 per case (8)
Invasive pneumococcal diseases (meningitis and pneumonia)	In the UK: ‐ More than 5,000 cases of IPD are diagnosed each year in England in all age groups (12) ‐ From July 2005 to June 2006, 797 cases of invasive pneumococcal disease in children <5 years (10)	In the UK: Meningitis: 2,274 £ per case (11) Post-meningitis sequelae: £3,335/year
External genital warts	In Europe: 600,000 new cases (14)	In France: €483 per case (payer perspective)
HPV-related cancers	In Europe: (46) ‐ Between 267,000 and 510,000 cervical precancerous lesions ‐ 35,000 cervical cancers ‐ 6,400 anal cancers ‐ 3,400 vulvar and vaginal cancers ‐ 1,300 penile cancers ‐ 11,000 head and neck cancers	In the UK: From £12,700 (penile cancer) to £16,400 (cervical cancer) per case (payer perspective) (13)
Zoster	In Europe: 1.7 new cases (54)	In Germany: €388 to €729 per case (payer perspective) (54)

For example, in November 1999, the United Kingdom was the first EU country to introduce mass vaccination against group C meningococcal disease (8). Invasive meningococcal disease is a serious bacterial infection mainly affecting young children. The disease progresses rapidly, has a fatality rate of 5–10%, and a considerable proportion of survivors have long-term disabling sequelae such as deafness, neurological impairments, and amputation. Since 2000, this vaccination programme has prevented over 9,000 cases of serious disease and more than 1,000 deaths (7). This represents considerable savings since the cost of treating these diseases was estimated at nearly £10 million, before vaccine introduction (8).

RV gastroenteritis (RVGE) is currently the most common cause of severe gastroenteritis in infants and young children in both developed and developing countries, leading to more than 87,000 hospitalisations per year in Europe (Table 1) (3). The direct yearly costs are estimated at almost €63 million in France and €67–80 million in Italy (5, 6). Two RV vaccines obtained market authorisation in Europe in 2008 and 2009. Belgium, Luxembourg, Austria, Finland, the UK, and more recently Germany have implemented universal RV vaccination. In the UK, the programme was launched in summer 2013 for children below 1-year-olds. The first full year's data on the impact of this national infant RV immunisation programme confirmed that the programme has been successful, with a 71% reduction in the number of cases. Significant reductions were also observed in numbers of General Practice (GP)-reported cases and in those reported by emergency departments (16).

Episodes of invasive pneumococcal pneumonia (IPD) in children make substantial demands on hospital healthcare and financial resources. For example, it was estimated that a child hospitalised for IPD in Spain had a median length of stay of 11.0 days, with an associated cost of €4,533 per stay. A substantial part of these costs can be avoided with a universal pneumococcal (PCV13) childhood vaccination programme and early management of complications (14).

A routine varicella vaccination programme could also have an important economic impact. It has been estimated that 68% of hospitalisations and 57% of deaths could be prevented with a 90% vaccination uptake in Italy (17). Vaccination costs would be more than offset by the reduction in varicella treatment costs within the first years after implementation. Despite this positive economical impact, varicella vaccine recommendations in Europe are heterogeneous, with only five countries where varicella vaccination is universally recommended for children at national level and two countries at regional level (18).

Influenza is a highly contagious disease that is responsible for 3–5 million cases of severe illness each year globally, with a high attack rate observed among children (9). A comprehensive systematic review, including 50 publications on the influenza burden in children in Europe, estimated that up to 20% of children aged 0–11 months with influenza are hospitalised with a mean length of stay of between 1.8 and 7.9 days (9). Therefore, successful implementation of these childhood vaccination programmes could lead to a substantial decrease in healthcare resource use, which should be balanced against the programme cost in the assessment of the programme efficiency.

### In adolescents and young adults

External genital warts (EGWs) are a sexually transmitted infection caused by various strains of human papillomavirus (HPV) that tend to infect particularly teenagers and young adults early after their sexual debut. It has been estimated that, in Europe, almost 600,000 new cases of EGWs occur each year (19).

The economic and human burden of EGWs is high (20). The average treatment cost and total annual direct cost of genital warts were estimated at €483 and €23 million, respectively, in France (21). The quadrivalent HPV vaccine protects against HPV types 16 and 18, as well as 6 and 11; these two latter types represent the causal pathogen for 90% of genital warts. A vaccination programme is, therefore, expected to have a significant impact on the occurrence of genital warts and their associated treatment cost. This assumption has been confirmed in countries such as Australia, which have implemented national vaccination programmes (22, 23).

A significant reduction in pertussis morbidity and mortality in infants and children has been observed where preschool and primary school pertussis vaccination has been implemented (24). However, the disease persists in infants who are too young to be vaccinated, and among adolescents and adults who have lost their disease- or vaccine-induced immunity against the disease or who were not vaccinated during childhood. The re-emergence of pertussis could be limited by a booster vaccination for adolescents and adults. Many countries (e.g., France, Germany, the USA, and Canada) have already integrated a pertussis booster for adolescents and/or adults into their current schedule. The results from a literature review showed that economical models converge towards the same conclusion, that is, that the pertussis booster vaccination is economically valuable when used in an appropriate context (24).

### In adults and elderly

Adult vaccination programmes have been slower to evolve in contrast to childhood vaccination programmes. It is necessary to fully appreciate the value of preventing these diseases to improve the uptake of adult vaccination.

A recent modelling study provided an illustration of the annual public health and economic benefits of influenza vaccination in Europe (25). Today, seasonal flu vaccination already imparts substantial annual health and economic benefits and prevents each year an average of 1.6–2.1 million cases, 45,000–66,000 hospitalisations, 25,000–37,000 influenza-related deaths, and €153–€219 million in healthcare costs (GP visits & hospitalization).

Seasonal influenza vaccination is recommended for approximately 180 million Europeans considered at risk (based on the WHO definition); however, only 44% of them (80 millions) actually receive an annual influenza immunisation, far from the 75% target coverage rate set by the EU Council Recommendation in 2009 (26). With the development of new and improved vaccines, additional benefits will be achieved in the future but today, full implementation of the 75% target coverage rate in the recommended target groups could immediately reduce the burden of seasonal influenza infections by an additional 1.6–1.7 million cases, 24,000–31,000 hospitalisations, 10,000–14,000 influenza-related deaths, and €77–€99 million of influenza-related additional healthcare costs saved yearly (25).

Until recently, influenza vaccines contained three influenza strains, two A strains, and one B strain. These strains are adapted every year to correspond to the circulating strains identified through the World Health Organisation's (WHO) surveillance system (27). There are two A strains, because two co-circulation of A strains have been observed in a given influenza season unlike, until recently, B strains for which only one strain was observed (28). However, in recent seasons the co-circulation of B strains has been increasingly observed and this has led to the WHO issuing recommendations to include four strains in seasonal influenza vaccines, that is, two A strains and two B strains (27). These new quadrivalent influenza vaccines (QIV) that are now commercially available can also play a role in the reduction of healthcare costs. An analysis conducted to quantify the potential public health impact of a QIV vaccine strategy compared with the current trivalent vaccine (TIV) strategy in the United States estimated that over 10 years (from 1999 to 2009), QIV could have prevented up to 2,741,575 cases of influenza, 21,440 hospitalisations, and 1,371 influenza-related deaths in the United States (29). Based on these estimates, it was estimated that over these 10 influenza seasons, QIV could have resulted in substantial cost savings of $3.1 billion to society and $292 million to third party payers if QIV had been used instead of TIV, at the same cost (30). Additionally, the burden and associated healthcare and societal costs from other vaccine-preventable diseases such as pneumococcal, herpes zoster, and pertussis are also substantial.

**Fig. 1:**
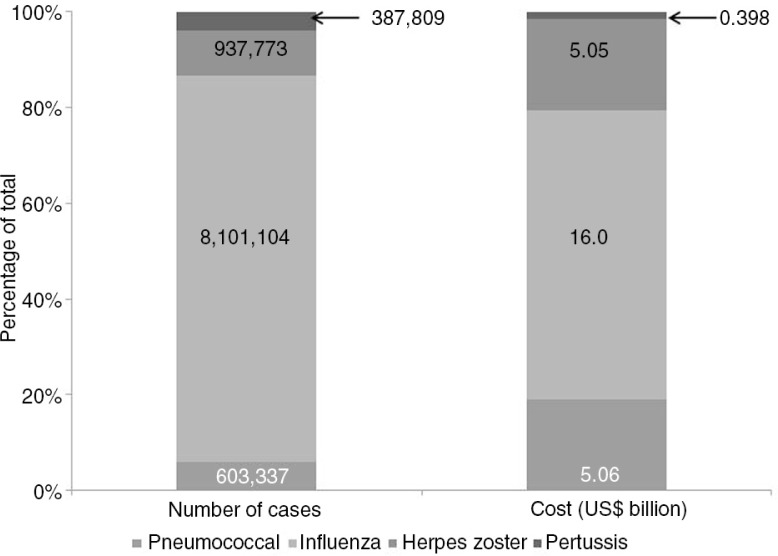
Incidence and total costs of four major adults vaccine-preventable diseases in the United States (2013).

It was recently estimated that the economic burden from four major adult, vaccine-preventable diseases (influenza, pneumococcal, herpes zoster, and pertussis) in those aged ≥50 years was more than 26.5 billion US$ (medical and indirect costs) in the United States in 2013 (31). Herpes zoster, influenza, and pneumococcal diseases were responsible for 19, 60, and 19% of these costs, respectively (Fig. 1) and medical costs accounted for 80, 91, 37, and 42% of total influenza, pneumococcal, zoster, and pertussis costs, respectively (31). Broadening adult vaccination programmes beyond influenza may, therefore, help reduce the overall burden of disease.

## Reduction in treatment prescription

Preventing diseases through vaccination also leads to a reduction in the consumption of medications. For example, medication use is high in children who have influenza; in addition to antipyretics and other symptomatic treatments, antibiotics were prescribed to 43% of children in primary care-based studies (9).

Vaccines that can prevent diseases, such as influenza, pneumococcal diseases, and shingles, in the elderly are likely to reduce not only the costs associated with medication consumption but also the costs associated with their side-effects. For example, a Dutch study of 84 patients with postherpetic neuralgia (PHN – a well-known complication of shingles) reported that 89% of patients took prescription medications such as antidepressants, opioids, various analgesics as well as antiepileptic medicines (32). The elderly are highly susceptible to side-effects from medications, partly because they generally take more than one medication, and there is often no dose adjustment (33). Polypharmacy is known to be associated with negative health outcomes and a major cause of drug interactions and safety problems in this age group (34).

## Prevention of nosocomial infections

Vaccination can also play a role in preventing nosocomial infections. For example, RV is one of the major aetiological agents for paediatric nosocomial diarrhoea, responsible for between 31 and 87% of cases (35). Nosocomial RVGE cases prolong hospital stays by 4–12 days and can lead to substantial additional costs (36). In Italy, costs resulting from nosocomial RV infection have been estimated at more than €8 million per year (37). Therefore, prevention of RV infection by vaccination could have a positive impact not only by reducing the number of children hospitalised for gastroenteritis, but also by reducing the number of nosocomial infections. The RV burden is significant and includes temporary reduction in the quality of children's lives, increased costs associated with the additional consumption of medical resources (increased length of hospital stay), and constraints on parents’/hospital staff's professional lives (38). A recent UK study reported that the likelihood of nosocomial infection with RV playing a significant role in children's hospital readmissions is high. This has important implications for hospital resources when considering costs and length of hospital stays (38).

*Clostridium difficile* infection (CDI) is also a major cause of nosocomial disease in western countries. CDI results in a significant burden not only for patients, through increased morbidity and mortality, but also for healthcare systems and society in general. On the basis of current incidence rates, the annual cost for the management of CDI is about $800 million in the United States and €3,000 million in Europe (39, 40). Pre-licensure clinical evaluation of the efficacy of two *C. difficile* vaccines is currently on-going. If the vaccines are found to be efficacious, this will provide hope for the future control of this costly bacterial infection.

Vaccines can also reduce the risk of secondary infections, which is relevant not just for those being vaccinated but the wider population. For example, influenza vaccination of healthcare workers (HCWs) has been shown to be associated with a substantial decrease in mortality for elderly patients (41). The cost of not vaccinating frontline HCWs is also significant in terms of missed benefits. It has been estimated that vaccinating each HCW in England and Wales against influenza would result in savings of £12 per vaccination (42).

## Prevention of cancer

In 2012, 2.7 million people were diagnosed with cancer in the European Union (EU27) (43). Nearly, one-fifth of all cancers in the world are caused by infectious agents, including viruses and bacteria. Among the most important infections associated with cancers are: HPVs that can cause most cervical and anal cancers as well as a fraction of oral cancers; hepatitis B virus (HBV) and hepatitis C virus (HCV) that can cause liver cancer; and *Helicobacter pylori* that is a bacterium that can cause stomach cancer (44).

Vaccines are the most effective way of preventing some of these infections. Effective vaccines against HBV have been available for several decades, and more than 90% of countries include HBV vaccination in their childhood immunisation programmes, which has been shown to be responsible for a dramatic reduction in liver cancer (45).

HPV vaccination is also highly effective in preventing infection with the HPV types that cause the majority of cervical cancers and other anogenital cancers in both women and men.

**Fig. 2:**
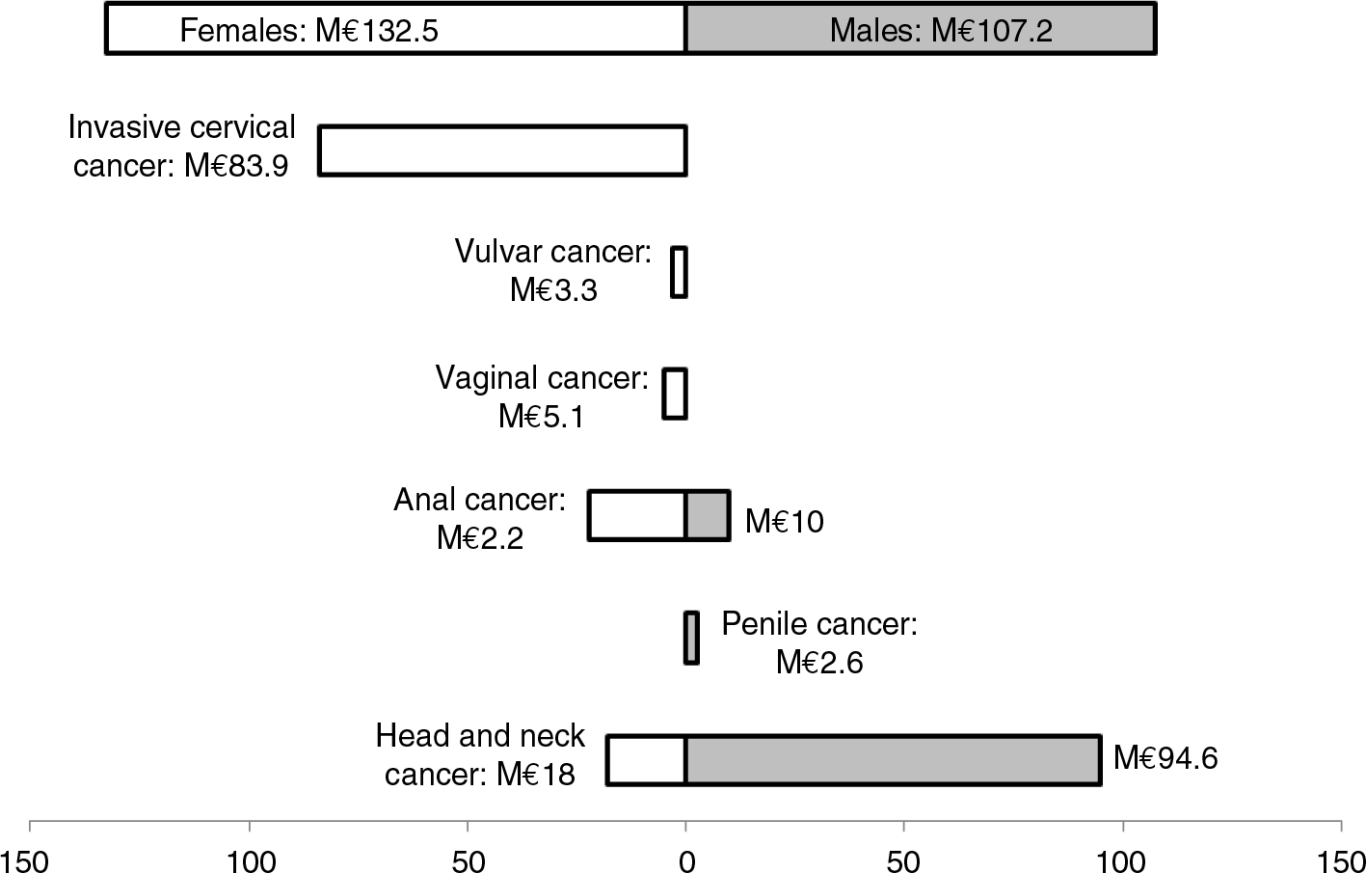
Estimated costs of HPV-associated cancers in France, payer perspective.

In Europe, between 67,000 and 80,000 new cancers attributable to HPV occur annually in women and men. These include penile and oropharyngeal cancers, for which HPV vaccines are not currently indicated (46, 15). Cervical cancer is the fourth most common cancer in women, diagnosed in approximately 34,000 women annually, and killing about 13,000 each year, in Europe (46). These cancers are responsible for a substantial economic burden in Europe with, for example, an estimated cost to the payers of €240 million in France in 2007 (Fig. 2) (47, 48). Additionally, a recent UK study reported that cervical cancer was not only a cost to the payer (47% of total costs) but also to the state (20% coming from annual loss of income tax and contributions) and to the women (31%) (49, 50).

Two HPV vaccines have been commercialised in Europe since 2006. It was estimated that vaccination of 12-year-old girls (70% uptake rate) would lead to a 86% reduction in HPV 16/18-related cervical cancers in females compared with screening alone (51). In addition to preventing cervical cancers and pre-cancerous lesions, HPV vaccination can reduce costs associated with borderline and mild dysplasia, and associated colposcopies. Primary prevention of cervical cancer by vaccination against high-risk HPV types has been an important development for public health. Currently, licensed HPV vaccines protect against HPV types that cause approximately 70% of cervical cancers. Results from a phase III clinical trial of a new nonavalent HPV vaccine are now available. This vaccine has the potential to expand the prophylactic cervical cancer coverage offered by current HPV vaccines from approximately 70 to 90% and the prevention of high-grade dysplasia from approximately 50 to 75–85%. (52). First cost-effectiveness analyses conducted in Canada concluded that the nonavalent vaccine will most likely represent a cost-effective alternative to the quadrivalent vaccine (53).

## Answering unmet medical needs

In addition to the direct impact on healthcare resources and costs, vaccines can improve the sustainability of healthcare systems by answering unmet medical needs. The herpes zoster vaccine is a good example, since there are no preventive measures or satisfactory treatments. Nearly 1.7 million cases of zoster are diagnosed in Europe each year (54). The management of herpes zoster, and its most frequent complication, that is, PHN, a chronic neuropathic pain that can persist for months or even years, is challenging, particularly in elderly people suffering from other chronic diseases and on polypharmacy (55). The direct costs of zoster and PHN for the healthcare service have been estimated to be more than €60 million per year in a country such as France, and these costs can be expected to increase with the steadily growing elderly population (56). This economic burden is likely to be underestimated as the estimate did not include additional costs for society, such as, loss of productivity, treatment for worsening underlying conditions, home help, and rehabilitation care (54).

## Conclusion

Although the access to some new vaccines is heterogeneous across Europe, they have been shown to reduce healthcare costs associated with hospitalisations and outpatient visits in several real life impact studies. Prevention of disease in children, adults, and the elderly through vaccination represents a unique opportunity to keep people healthy and outside of the healthcare system. Hence, vaccination can contribute to the sustainability of healthcare systems by avoiding unnecessary use of financial and human resources and freeing resources for other medical interventions. Improving uptake of vaccination programmes is critical in periods when governments are looking for solutions for more efficient healthcare resource use. Thus, widespread health promotion and disease prevention are key factors for the long-term sustainability of health systems.
